# Post-Fire Characteristics of Concrete Beams Reinforced with Hybrid FRP Bars

**DOI:** 10.3390/ma13051248

**Published:** 2020-03-10

**Authors:** Kostiantyn Protchenko, Elżbieta Szmigiera

**Affiliations:** Department of Civil Engineering, Warsaw University of Technology, 1 00-661 Warsaw, Poland; e.szmigiera@il.pw.edu.pl

**Keywords:** fiber-reinforced polymers, FRP bars, HFRP, fire resistance of FRP, beams reinforced with FRP, fire testing of FRP reinforced members, nano-HFRP bars

## Abstract

One of the main concerns of experimental and numerical investigations regarding the behavior of fiber-reinforced polymer reinforced concrete (FRP-RC) members is their fire resistance to elevated temperatures and structural performance at and after fire exposure. However, the data currently available on the behavior of fiber-reinforced polymer (FRP) reinforced members related to elevated temperatures are scarce, specifically relating to the strength capacity of beams after being subjected to elevated temperatures. This paper investigates the residual strength capacity of beams strengthened internally with various (FRP) reinforcement types after being subjected to high temperatures, reflecting the conditions of a fire. The testing was made for concrete beams reinforced with three different types of FRP bars: (i) basalt-FRP (BFRP), (ii) hybrid FRP with carbon and basalt fibers (HFRP) and (iii) nano-hybrid FRP (nHFRP), with modification of the epoxy matrix of the rebar. Tested beams were first loaded at 50% of their ultimate strength capacity, then unloaded before being heated in a furnace and allowed to cool, and finally reloaded flexurally until failure. The results show an atypical behavior observed for HFRP bars and nHFRP bars reinforced beams, where after a certain temperature threshold the deflection began to decrease. The authors suggest that this phenomenon is connected with the thermal expansion coefficient of the carbon fibers present in HFRP and nHFRP bars and therefore creep can appear in those fibers, which causes an effect of “prestressing” of the beams.

## 1. Introduction

For the last few decades, the use of fiber-reinforced polymers (FRP) composites for concrete structures has experienced explosive growth. A major reason of its implementation lies in the unique characteristics of these materials and their constituents, as well as the appropriate use of FRP composites for its considered design purpose. Numerous investigations have shown that FRP composites are effective materials for use in concrete members [1,2].

The application of FRP bars as internal reinforcement in concrete members has many advantages, as opposed to structural members reinforced with steel bars [3]. Durability, strength and stability are the main criteria when selecting a material in the design of concrete structures with internal FRP reinforcement [4,5,6,7]. 

The progress in manufacturing technology has introduced new types of FRP bars, and furthermore hybrid FRP (HFRP), which allow one to adjust FRP characteristics to a specific design situation. HFRP bars contain several types of reinforcing constituents and/or several types of adhesive constituents in a matrix. Moreover, some authors suggest that such hybridization of the constituents of FRP bars can prevent changes in their behavior, making it semi-ductile instead of linear [8,9].

Therefore, their use in the form of FRP bars as a replacement of conventional reinforcement for reinforced concrete (RC) structures has become attractive. In addition, these materials are unaffected by electrochemical deterioration [9,10].

FRP composites consist of unidirectional organic or inorganic fibers embedded in a thermosetting or thermoplastic polymer matrix [9]. The properties of FRP composites are dependent on the properties of their constituents and the relative proportions of the fiber, known as the fiber-volume ratio [11]. The matrix can be seriously affected at elevated temperatures, therefore it is necessary to examine the behavior of bars subjected to fire exposure, as well as structures reinforced with these materials. Currently, the use of FRP reinforcement for RC structures is limited and only includes cases when fire resistance aspects are not particularly meaningful.

Building structures must satisfy the requirements of building codes, including requirements related to the behavior of structures in a fire. Fire ratings for buildings refer to the amount of time a structure can be exposed to fire before the structure collapses. The most relevant property of FRP bars is not its flammability or its reaction to fire, but rather its ability to continue to sustain loads in an environment of rapidly rising temperature [12,13].

The mechanical performance of FRP reinforcement is already known at ambient temperatures, however, several technical characteristics of FRP bars make designing structures with FRP reinforcement different from conventional RC design in regard to fire resistance aspects [11]. The data available from standards on the behavior of FRPs and FRP reinforced concrete (FRP-RC) members at elevated temperatures are scarce. ACI committee ACI 440.1R-15 [14] describes the material characteristics needed to design the minimum reinforcement in the perspective of temperature and shrinkage thresholds. The Canadian code CAN/CSA S806-12, Annex R for slabs [15] provides a design procedure in a fire situation based on critical temperatures related to the FRP bars [16].

The material properties and behavior of FRP composites at elevated temperatures is a critical topic that needs clear understanding in terms of their structural uses in civil engineering. Some studies state that high temperatures can reduce the mechanical properties of FRP composites significantly. For instance, Wang et al. [17] investigated the mechanical properties of Carbon-FRP (CFRP) plates, and it was discovered that when the temperature of the CFRP plates reached 300 °C, the ultimate strength of the plates became approximately 50% of their room-temperature strength. In addition, the tensile strength of the plate became as low as 7% of the room-temperature tensile strength at the approximate peak temperature of 700 °C. Furthermore, Hu et al. [18] investigated the reduction in the properties for different directions (0°, 45° and 90° of the load-to-fiber angle) for basalt-FRP (BFRP) laminates. Based on the outcomes, it was found that the elasticity modulus in the various directions was reduced to a very low level (less than 20%) from 20 to 250 °C. Furthermore, the in-plane shear modulus of BFRP at 250 °C was only 3% of that at 20 °C.

As mentioned in the literature, a reduction in strength and bond properties starts when the temperatures become close to the glass transition temperature, *Tg*, are reached [19]. The magnitude of *Tg* depends on the type of resin and ranges between 70 °C and 175 °C [20], however for some of the resins, glass transition temperature can be more than 500 °C. However, the decisive factors of the strength deterioration are also fiber type, manufacturing process and the quality of FRP bars and, above all, the temperature attained in FRP [21]. Therefore FRP-RC structures can withstand fire much better, due to the fact that concrete provides reliable protection for FRP bars.

Furthermore, the literature provides experimental results from failure tests performed on FRP reinforced concrete slabs [22,23,24,25] and beams [26,27,28], working in flexure that were exposed to conventional fire conditions. Abassi and Hogg [12] investigated the fire resistance of concrete beams reinforced with Glass-FRP (GFRP) bars with a concrete cover of about 70 mm. The beams retained their fire endurance for over 90 min, therefore a minimum concrete cover of 70 mm was recommended for the design of GFRP-RC beams under fire action.

However, the outlined literature shows a gap in research on the topic of the post-fire behavior of FRP-RC structures [24]. Nigro at al. [23] investigated the residual strength capacity for slabs reinforced with GFRP bars. Two unloaded slabs, after being heated, attained deflections of approximately 70 mm, due to their own weight and thermal strains, and their residual resistance, evaluated after the cooling phase, which was about 55% to 100% of the ultimate design bearing moment resistance. Moreover, it was stated that the typical values of concrete clear cover (i.e., between 30 and 50 mm) can be adopted.

On the other hand, the relatively non-corrosive behavior of the fibers prevents a significant reduction of concrete clear cover without compromising the material’s durability [28,29,30]. This enables thinner structures to be built, whose construction requires less raw materials and is more sustainable, environmentally friendly, durable, and cost-efficient. However, thin-walled elements with thin concrete covers may subject the reinforcement to substantial effects when exposed to elevated temperatures [31]. 

As known from experiments on beams reinforced with steel reinforcement, the concrete clear cover, and the fire scenario have the most significant influence on the response of the concrete element subjected to fire [32]. Nevertheless, in the design of FRP-RC structures, the reinforcement type and obtaining of a specific high-temperature resistance for FRP bars is one of the main concerns. The use of FRP in structures vulnerable to fire needs further research since this is one of the main reasons that limit the widespread use of FRP in construction. 

However, the behavior of such reinforcement during a fire is still unknown. In addition, the mentioned literature demonstrates a lack of insight on the behavior of basalt FRP (BFRP) and HFRP reinforcement at and after applying fire conditions. Therefore, the authors attempted to investigate the post-fire behavior of RC flexural members reinforced with these types of reinforcement. The FRP-RC members were not subjected to loading during heating phase to allow to concentrate on the performance of FRP bars after being subjected to elevated temperatures. Different reinforcement configurations were examined for the tension zone to check the influence of different types of the bars.

This paper investigates the residual capacity of full-scale FRP-RC beams after being subjected to elevated temperatures. For residual testing, the beams were preliminary loaded with a force of 50% of their ultimate strength capacity (checked at room temperature), then the beams were unloaded. Afterwards the beams were placed into a furnace and heated for approximately one hour. After heating, the beams were allowed to cool, and finally reloaded flexurally until failure.

The results of the investigations were discussed, with particular emphasis on the structural performance of the concrete members and on the types of FRP bars used. 

## 2. Novelty and Purpose of the Work

Tested specimens were subjected to specific fire actions, where the midsection, approximately 1/3 of the length of the beams, was exposed to heating from both the bottom and the sides. In conventional testing, the entire span length of concrete members is usually subjected to heating and loading is applied simultaneously. The presented investigation is focused on residual fire resistance testing, where the samples were heated and the strength capacity was checked afterwards.

As one of the main aims was to examine the influence of different reinforcement types, the testing was made for concrete beams reinforced with three different types of FRP bars, including newly developed hybrid FRP bars: (i) basalt-FRP (BFRP), (ii) hybrid FRP with carbon and basalt fibers (HFRP) and (iii) nano-hybrid FRP (nHFRP), with modification of the epoxy matrix of the rebar.

## 3. The Concept of Hybrid FRP Bars

The concept behind the invention of HFRP bars came as a desire to obtain a material with improved and adjustable properties, where the final product can exploit the most favorable qualities of every individual constituents.

In the context of this work, the term “hybridization” should be understood as a physical substitution of some of the fibers by another type. The proposed constituents for these hybrid bars: carbon and basalt fibers, were embedded in epoxy resin creating HC/BFRP (HFRP) bars.

Processing HFRP bars is analogous to BFRP bars, a certain amount of the basalt roving is physically replaced with carbon roving during the pultrusion process; subsequently both were embedded in epoxy resin. Different constituents and their location were estimated. Basalt and carbon fibers of low strength (LS) type were selected due to their similar strain parameters. Different volume fractions for carbon and basalt (C:B) fibers were considered as well, before production of the bars. 

Analytical and numerical estimations were performed, prior to the production of HFRP and nHFRP bars. The properties of composite bars in the longitudinal direction can be calculated using the rule of mixtures (ROM) (Voigt model), as it comes from the literature [33,34,35]. The transverse properties can be obtained with Halpin–Tsai and other semi-empirical models [36,37]. However, these formulas do not consider bars configuration, i.e., the location of fibers, so, it was agreed to prepare an additional numerical simulation for this purpose.

The numerical simulation of tensile strength test for HFRP bars with different configurations of fibers was performed by finite element methods (FEM) in the software ANSYS^®^ Academic Research Mechanical, Release 16.2 (Ansys Inc., Canonsburg, PA, USA) [38]. Two different bar configurations were proposed, one where carbon fibers substituted the basalt fibers in the core region, the other one with carbon fibers located in the near-surface region.

The bars were modelled as cylindrical elements with a diameter of 8 mm and a length of 850 mm. A constant pressure of 500 MPa was applied on the side edges. One central point was fixed in the *y* and *z* directions (non-longitudinal directions). The structure of the HFRP bars consisted of a core and surface region, which were perfectly interconnected.

The obtained results from numerical modelling were compared with analytical considerations and are found to be in a good convergence with each other. The results for different fibers arrangement and different volume fractions of fibers are described in Table 1. The comparison between analytical considerations and numerical simulations of FRP bars was explained in more detail in [8].

The results showed that bar configuration is less important than the volume fraction of fibers. The difference between various bar configurations can be a maximum of 2%, meanwhile, the volume fraction of all analyzed configurations can influence the final stiffness by 74.6%.

Several technological issues were observed while placing the carbon fibers located in the near-surface region, including increased heterogeneity at fiber locations and local scorching of bars caused by temperature increases. Consequently, the most suitable location for carbon fibers is in the core region of the HFRP rebar.

In addition, nHFRP bars were prepared with an addition of nano-silica fillers to the resin consistency. The additives were used for improving the chemical cohesion between the agents of nHFRP and to improve the overall properties of the bars, as suggested in some sources [39,40,41]. 

**Figure 1:**
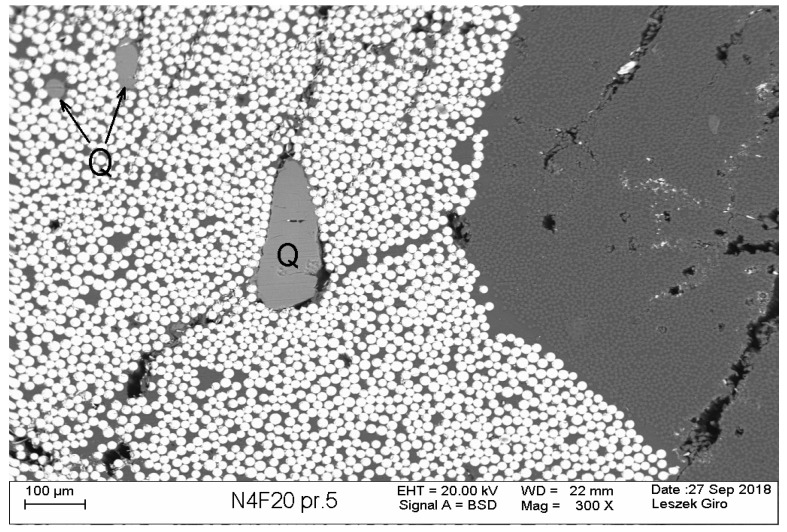
SEM images of nano-hybrid fiber-reinforced polymer (nHFRP) rebar with 4 mm diameter, after cooling phase (till-20 °C). Detailed properties for the selected bars are represented in subchapter Section 4.1.

Figure 1 represents an example of scanning electron microscope (SEM) images, showing the constituents of nHFRP bars, where it is possible to distinguish different fibers and nano-additives (by their dimensions and color). Visible areas of nanosilica locations are marked as *Q*, bright spots indicate basalt fibers, while carbon fibers appear as darker areas. 

## 4. Experimental Program

The experimental program is involved in the design and fabrication of the six FRP reinforced concrete beams without any fire protection system. The investigation consisted of fire tests performed on three simply supported concrete beams strengthened internally with different reinforcement in the tensile zone (Set 1) and three corresponding reference beams that were used for comparison (Set 2).

The beams of Set 1 were preliminarily loaded by a force of 50% of their ultimate strength capacity to simulate realistic situations, i.e., the appearance of cracks. As for the next step, unloaded cracked beams were placed into a furnace in such a way that the mid-section of the beams was inside it. After the cooling phase, the beams’ capacity was checked with a four-point flexural test. The beams of Set 2 were tested with four-point flexural tests without preliminary loading and were not exposed to elevated temperature. Table 2 shows the description of specimens used in the tests.

### 4.1. Materials

#### 4.1.1. Concrete

The concrete mixture design was identical for all beams: a typical concrete mix C40/45 was used. Ordinary Portland cement CEM III/A, ash, and crushed stone (silica), with a nominal maximum size of 16 mm, were used in concrete mixes. The beams were cured for a complete 28-day period in the lab, before they were moved to the testing frame. The cubic specimens from the same concrete mixture were checked to confirm the concrete class.

#### 4.1.2. Bars

A more detailed description for choosing the configuration of the bars and their characteristics is reported in the following companion papers [42,43,44]. For these tests, the bar types BFRP, HFRP, and nHFRP were chosen. 

The selected volume fractions of carbon-to-basalt fibers for HFRP bars were assumed as 1:4 (i.e., 16% of carbon fibers, 64% of basalt fibers and 20% of epoxy resin). For the nHFRP bars, the matrix was modified by adding a four-component 1300 System^®^ (CIECH Sarzyna S.A., Nowa Sarzyna, Poland) to the epoxy resin. A sol with the nanosilica particles with a concentration of 25 to 30% by weight was used. Average diameter of particles was equal 24.7 nm, containing two fractions: fine (80%) and coarse (20%).

The mean values of maximum strength, *F_u_*, limit stress, *f_u_*, modulus of elasticity, *E_11_*, and the limit strain, *ε_u_*, for BFRP, HFRP and nHFRP bars were obtained from tensile tests and are shown in Table 3.

### 4.2. Specimen Dimensions and Reinforcement Configuration

All specimens had a rectangular cross-section with dimensions 140 mm wide and 260 mm high and were 3200 mm long. The clear cover, *c_nom_*, was equal to 30 mm from all sides for all tested specimens.

**Figure 2:**
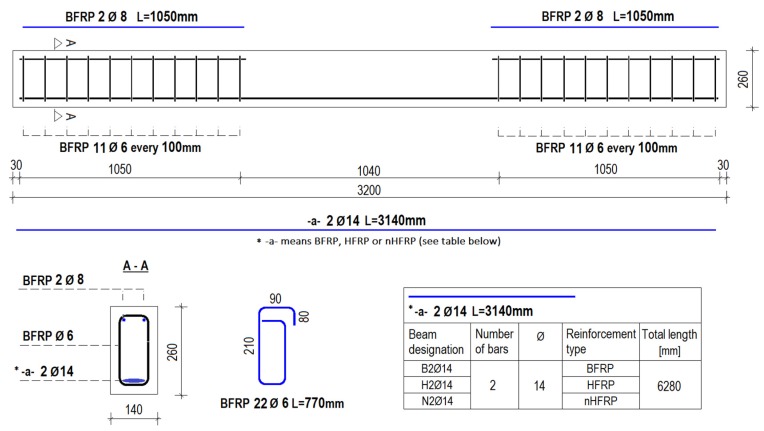
Schematic details of beam specimens.

The top reinforcement (compression zone) and shear reinforcement (stirrups) were kept constant for all beams. Two BFRP bars with a diameter of 8 mm were used as longitudinal top reinforcement. The stirrups made of BFRP with a diameter of 6 mm were used as shear reinforcement. The stirrup spacing was assumed as 100 mm and the mid-part of the beam did not have stirrups to simulate clear bending. The bottom reinforcement (tension zone) was a variable parameter and described based on the reinforcement type, as mentioned in Figure 2. Figure 2 shows the scheme of tested specimens.

### 4.3. Test Setup

Prior to casting, each specimen was instrumented with thermocouples of Type K (till 1200 °C) inside the specimens at different locations, in order to monitor the temperature during fire exposure. Eight thermocouples were used for each beam that were embedded at different depths. Seven of the thermocouples were added into the concrete and one of the thermocouples, *T3*, was embedded on the upper surface of a bar.

Three dial gauges were applied to the top face in order to measure the deflections in the mid-section of the beams. In addition, two dial gauges were added on the sides to measure possible beam extensions.

The specimens were exposed to heat in the mid-section from below and from the sides. Outputs were recorded every half second during the heating and cooling process. 

**Figure 3:**
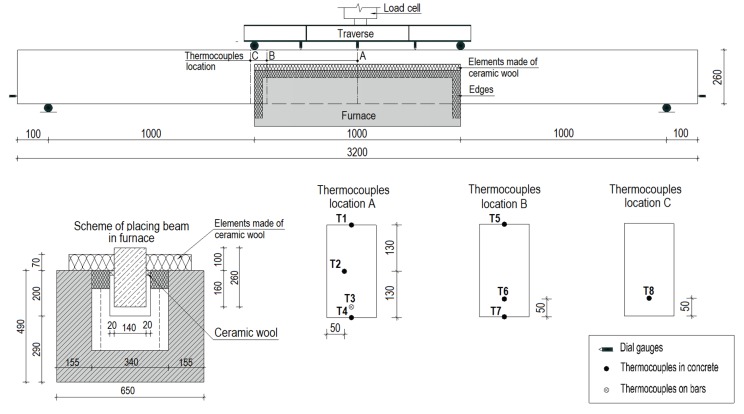
Test setup description.

Before the temperature was applied, the beams from Set 1 were loaded to 50% of their ultimate strength load (as described in Table 1). Set 2 was tested without temperature exposure and preliminary loading. Figure 3 shows the test setup of the beams.

Beams were heated from the bottom, as well as parts of both sides, for a period of approximately one hour. The standards provide the means of heating beams from room temperature to temperatures representative of a fire, taking into account a time parameter. Structural fire testing was performed under standard conditions in accordance with norms [45] and a standard heating curve ISO-834 (1999) [46], represented by formula:

*T_ISO_* = *T_0_* + 345 × log(8*t* + 1), (1)

where: *T_ISO_* is the temperature (°C), *T_0_* is the room temperature (assumed to be 20 °C) and *t* is the time (min).

**Figure 4:**
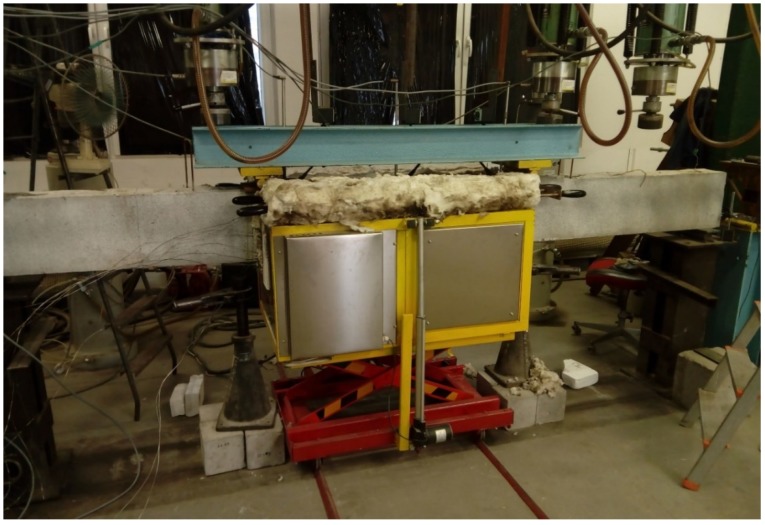
Test setup during heating phase.

Figure 4 illustrates the test setup during the heating phase.

## 5. Results and Discussion

**Figure 5:**
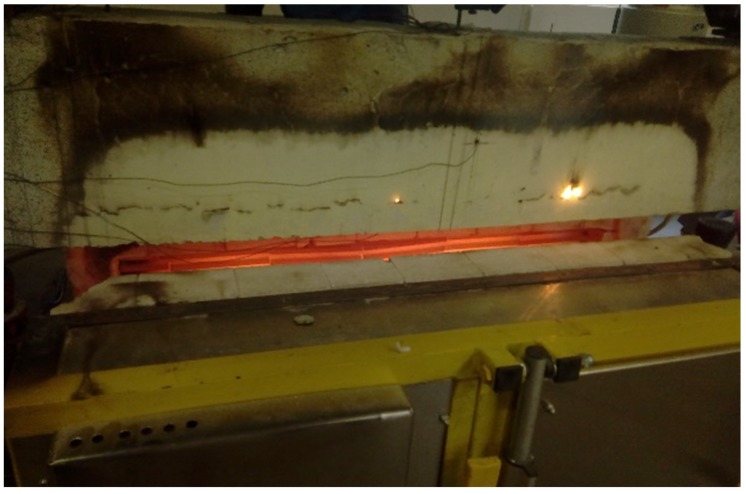
Sample B2Ø14 just after being taken out of the furnace.

The performed tests provide a means to estimate the influence of elevated temperatures on beams reinforced with FRP and therefore investigate their thermal behavior being subjected to those temperatures. All specimens showed a typical flexural-ductile failure, the beams of Set 1 were destroyed due to reinforcement failure in the tensile zone, however the reason of the beams destruction of Set 2 was the concrete crushing in the compression zone. Figure 5 demonstrates the beam sample B2Ø14 from Set 1 just after it was taken out of the furnace.

**Figure 6:**
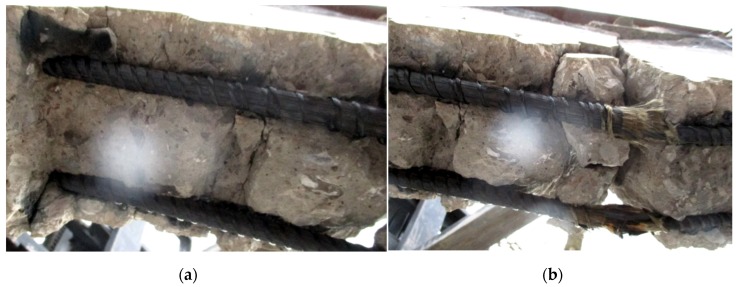
The destruction of sample H2Ø14: (**a**) gaps between bars and concrete surface; (**b**) reinforcement failure.

As shown on Figure 6, after the hybrid FRP bars were uncovered by removing the clear cover, the temperature caused a burning of the FRP bars. This resulted in the evaporation of the matrix in the middle of the bars. The temperature caused a de-bonding with the concrete, completely separating the reinforcement from the concrete (Figure 6a). However, a major portion of the fibers remained in the same place and continued to sustain the load (Figure 6b).

**Figure 7:**
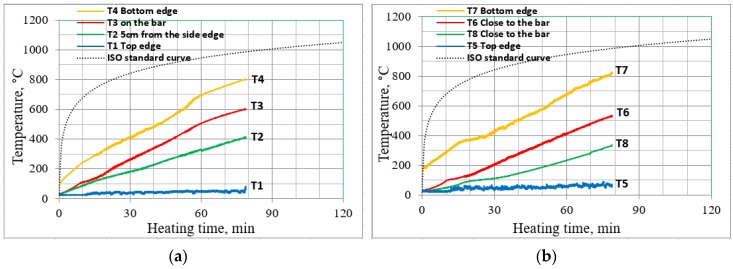
Measurements from the thermocouples embedded in the sample B2Ø14; (**a**) location A; (**b**) location B and C.

Analyses of the temperatures attained at different depths were possible with the collection of measurements from eight thermocouples embedded in the concrete and on the bars. An example temperature distribution is shown in Figure 7a,b, which demonstrate the measurements obtained from thermocouples for sample B2Ø14. The thermocouples are numbered in accordance with the scheme (Figure 3) and represent temperatures at different locations.

From Figure 7, it can be seen that the temperatures obtained at thermocouples *T4* and *T7* are similar, indicating that the temperatures were distributed uniformly. At the same time, measurements obtained from thermocouples *T1* and *T5*, indicate that the gaps between the beam interfaces and edges of the furnace were carefully insulated with ceramic and rock wool. In addition, it can also be seen that the maximum temperatures measured at the bottom edge were approximately 20% lower (at the last point) than the applied temperatures.

**Figure 8:**
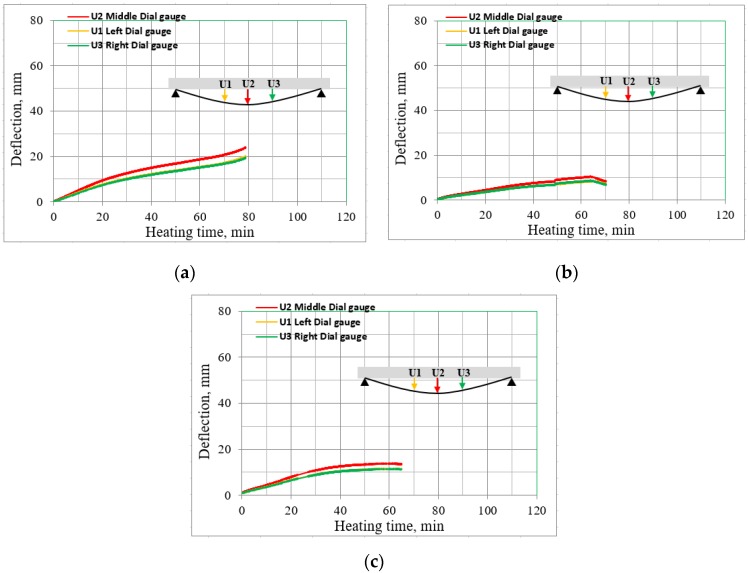
Mid-span deflections during the heating phase; (**a**) B2Ø14, (**b**) H2Ø14, (**c**) N2Ø14.

The sample beam B2Ø14 from Set 1 showed a time-vertical deflection relationship more similar to a typical one for steel reinforced concrete beams [13,32]; the deflections were only increasing. Despite the larger initial deflections for beam samples B2Ø14, H2Ø14 and N2Ø14, the increase of deflections during fire exposure started to be twice lower than in the case of analogous beams with steel reinforcement. However, samples H2Ø14 and N2Ø14 demonstrated an atypical behavior for beams subjected to elevated temperatures, since at a certain point the deflection began to decrease. At that time, the registered temperature on the bar surface was in the range of 550–570 °C. Figure 8 describes the deflections obtained by three dial gauges during the heating process of the beams from Set 1.

**Figure 9:**
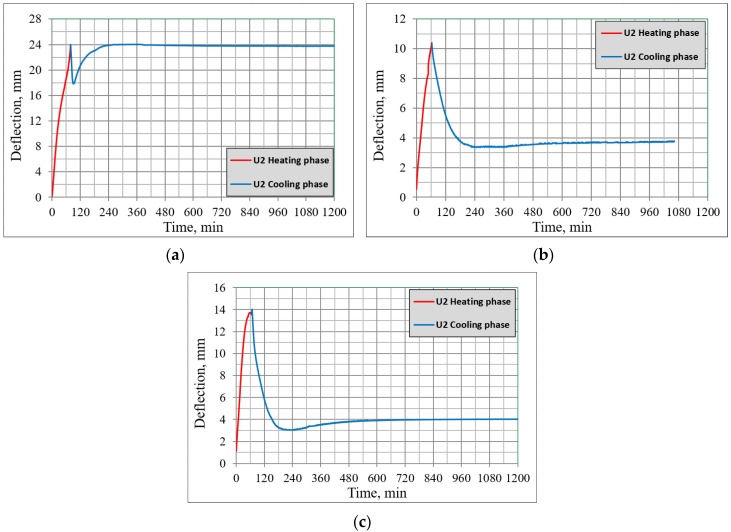
Mid-span deflections during the heating and cooling phases; (**a**) B2Ø14, (**b**) H2Ø14, (**c**) N2Ø14.

Figure 9 shows the deflections of beams from Set 1, measured by the middle dial gauge, *U2*, during the heating and cooling phases. It was noticed that just after the heating phase, the deflection of sample B2Ø14 at first started to decrease and then returned to its maximum value (shown in the Figure 9a), however for both samples H2Ø14 and N2Ø14 the deflections during the cooling phase started to decrease until approx. 30% of their maximum value was reached (Figure 9b,c). In the opinion of the authors, this difference is due to the carbon fiber presence in hybrid FRP bars, which works as a kind of “prestressing” during heating (at certain thresholds as shown in Figure 8), as well as during the cooling process, since beam displacement becomes smaller and then remains constant.

**Figure 10:**
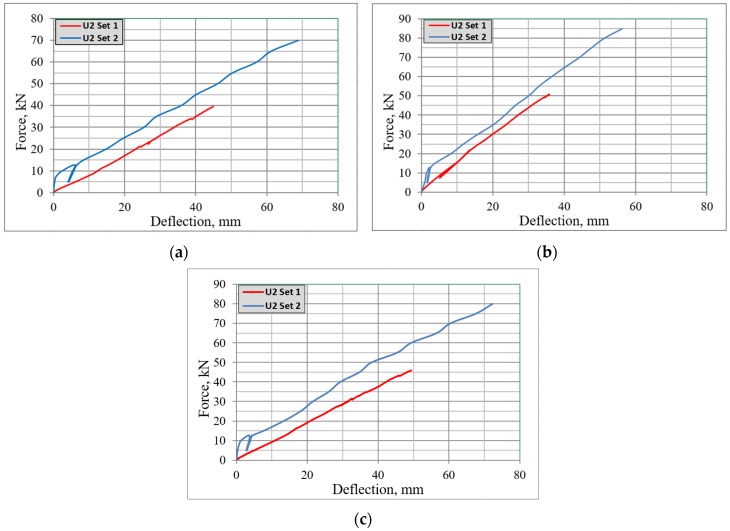
Comparison of the ultimate capacity for beams from Set 1 and Set 2; (**a**) B2Ø14, (**b**) H2Ø14, (**c**) N2Ø14.

The strength capacity for beams of Set 1 was compared with beams of Set 2, which were tested without the influence of elevated temperatures (Figure 10). The beams from Set 2 were only subjected to loading in the four-point flexural test, and subsequently had a greater strength capacity. The samples of Set 2 were loaded till 12.5 kN and then loading was reduced to 5 kN, after one more analogous cycle of loading the beams until failure. The cyclic loading was applied in order to reduce effects of plastic strains. 

A comparison between results of tested beams is represented in Table 4. 

Set 2 (*F_u, reference_*) showed a higher strength capacity by 43%, 40% and 43% respectively for beams B2Ø14; H2Ø14 and N2Ø14 from Set 1 (*F_u, tested_*). A comparison between results for beams from Set 1 and Set 2 is shown in Figure 10. The highest strength capacity that was obtained by the tested sample H2Ø14 for both beams was equal to 51 and 85 kN for beams from Set 1 and Set 2, respectively.

## 6. Conclusions

This study investigates the residual strength capacity for beams subjected to elevated temperatures that were preliminarily loaded by half of their ultimate capacity. Solely FRP reinforcement was used; beams were having different reinforcement in the tensile zone (bottom part of the beams): BFRP, HFRP or nHFRP bars. Reinforcement used in the compression zone and stirrups were made from BFRP.

Obtained results show that deflections obtained from current investigation for beams reinforced with FRP bars were much smaller than the results received for similar beams reinforced with steel bars in other studies [13,32]. In addition, the behavior of FRP-RC beams was quite uncharacteristic when subjected to elevated temperature, as well as during the cooling phase. 

Based on the experimental testing of beams reinforced with FRP, the following main conclusive remarks can be drawn:

1. All tested samples subjected to elevated temperatures were destroyed due to the tensile zone reaching its ultimate strength capacity. The authors suggest that this issue can be related to the reduction of mechanical properties experienced by the bars after being subjected to fire actions.
2. The overall strength capacity of the FRP reinforced beams after being subjected to fire exposure was reduced; by approx. 43% for beams with the tensile zone reinforced with BFRP bars, 40% and 43% for beams reinforced with HFRP and nHFRP bars, correspondingly.
3. The highest strength capacity was obtained by beams reinforced with HFRP bars. The strength capacity of the beams reinforced with HFRP bars after applying elevated temperatures was reduced by approximately 40% and was equal to 51 kN. As it can be seen from the force-deflection plots; the post-fire behavior of FRP-RC beams (Set 1) was similar to beams not subjected to fire exposure (Set 2) until failure. Nevertheless, the reduction in strength is significant.
4. When the temperature on the bars reached the range of 550–570 °C, and the bottom edge of beams were heated to around 700 °C, the deflections of the beams reinforced with hybrid FRP bars, i.e., for HFRP and nHFRP reinforced beams) started to decrease. This behavior was different for the BFRP reinforced beam, which was more similar to typical steel reinforced beams.

Similar behavior was observed during the cooling phase. It was noticed that deflections obtained for beams reinforced with BFRP bars were reduced by 40% initially and then returned to their maximum value of 25 mm during the cooling phase. The deflections for the beams reinforced with hybrid FRP bars were decreasing and then stopped at approx. 30% of their maximum value; 4 mm during the cooling phase. Since the deflections became smaller for beams reinforced with HFRP bars, the authors suggest that it relates to the presence of carbon fibers, which cause a kind of “prestressing” for the beams at a certain temperature threshold, as well as during the cooling phase.

The authors want to conclude that further research is still needed to estimate the influence of elevated temperatures on the strength capacity of FRP-reinforced beams, with taking into account other variables such as the reinforcement type and the type of fire scenario, which can significantly change the mechanical behavior of RC structures strengthened with FRP reinforcement. Therefore, other analyses with regard to reinforcement types and their ratios will be made.

## Figures and Tables

**Table 1:** Comparison of Young’s modulus obtained from rule of mixtures (ROM) and finite element methods (FEM) modeling.

Volume Fraction Dependence C:B ^1^	Modulus of Elasticity of Hybrid Fiber-Reinforced Polymer (HFRP) Bars, GPa		
	ROM The Location of Fibers Is Neglected	FEM Carbon Fibers Location	
		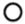	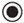
1:9	83.3	83.0	82.9
1:4	94.8	93.6	93.6
1:3	100.5	101.1	100.1
1:2	110.0	110.5	109.1
1:1	129.1	127.8	129.2

^1^ C—carbon fibers; B—basalt fibers.

**Table 2:** Loading and description of specimens. BFRP = basalt fiber-reinforced polymer.

Set No.	Beam Designation ^1^	Reinforcement Ratio	Reinforcement Type (Tension Zone)	Preliminary Loaded (50% of the Ultimate Load)
-	-	(%)	Number/dia/type	(kN)
1	B2Ø14	0.98	2/14/BFRP	30
	H2Ø14		2/14/HFRP	40
	N2Ø14		2/14/nHFRP	40
2	B2Ø14		2/14/BFRP	0
	H2Ø14		2/14/HFRP	0
	N2Ø14		2/14/nHFRP	0

^1^ BFRP—basalt fiber-reinforced polymers; HFRP—hybrid fiber-reinforced polymers; nHFRP—nano hybrid fiber-reinforced polymers.

**Table 3:** Mechanical properties of the fiber-reinforced polymer (FRP) bars utilized in the tests.

Type of Bars Type/Dia	Maximum Tensile Force *F_u_* (kN)	Tensile Strength *f_u_* (MPa)	Tensile Strength at Rupture *ε_u_* (%)	Modulus of Elasticity *E_11_* (GPa)
BFRP Ø6	37.07	1148.81	2.48	46. 47
BFRP Ø8	60.03	1103.30	2.52	43.87
BFRP Ø14	179.26	1101.94	2.39	46.02
HFRP Ø14	206.57	1160.06	1.61	72.12
nHFRP Ø14	150.54	958.00	1.58	60.44

**Table 4:** Mechanical properties of the FRP bars utilized in the tests.

Type of Bars	BFRP		HFRP		nHFRP	
	*F_u, tested_* (kN)	*F_u, reference_* (kN)	*F_u, tested_* (kN)	*F_u, reference_* (kN)	*F_u, tested_* (kN)	*F_u, reference_* (kN)
Ultimate force	40.00	70.00	51.00	85.00	46.00	81.00
*F_u,tested_/F_u,reference_* (%)	57		60		57	
